# Sex Differences in Human Olfaction: A Meta-Analysis

**DOI:** 10.3389/fpsyg.2019.00242

**Published:** 2019-02-13

**Authors:** Piotr Sorokowski, Maciej Karwowski, Michał Misiak, Michalina Konstancja Marczak, Martyna Dziekan, Thomas Hummel, Agnieszka Sorokowska

**Affiliations:** ^1^Institute of Psychology, University of Wroclaw, Wroclaw, Poland; ^2^Institute of Psychology, Adam Mickiewicz University in Poznan, Poznan, Poland; ^3^Interdisziplinäres Zentrum Geruch und Geschmack, Universitätsklinikum Carl Gustav Carus, Dresden, Germany; ^4^Klinik und Poliklinik für Psychotherapie und Psychosomatik, Universitätsklinikum Carl Gustav Carus, Technische Universität Dresden, Dresden, Germany

**Keywords:** olfaction, sex differences, meta-analysis, SST, UPSIT

## Abstract

Although the view that women's olfactory abilities outperform men's is taken for granted, some studies involving large samples suggested that male and female olfactory abilities are actually similar. To address this discrepancy, we conducted a meta-analysis of existing studies on olfaction, targeting possible sex differences. The analyzed sample comprised *n* = 8 848 (5 065 women and 3 783 men) for olfactory threshold (as measured with the Sniffin Sticks Test; SST), *n* = 8 067 (4 496 women and 3 571 men) for discrimination (SST), *n* = 13 670 (7 501 women and 6 169 men) for identification (SST), and a total sample of *n* = 7 154 (3 866 women and 3 288 men) for works using University of Pennsylvania Smell Identification Test (UPSIT). We conducted separate meta-analyses for each aspect of olfaction: identification, discrimination and threshold. The results of our meta-analysis indicate that women generally outperform men in olfactory abilities. What is more, they do so in every aspect of olfaction analyzed in the current study. However, the effect sizes were weak and ranged between *g* = 0.08 and *g* = 0.30. We discuss our findings in the context of factors that potentially shape sex differences in olfaction. Nevertheless, although our findings seem to confirm the “common knowledge” on female olfactory superiority, it needs to be emphasized that the effect sizes we observed were notably small.

## Introduction

Olfaction can strongly influence human behavior (McGann, 2017), and smells can have numerous effects on the human nervous system—for example, they can modulate heart rate variation (Brauchli et al., 1995), or subjective evaluation of pain (Bartolo et al., 2013). Some specific odors can also increase vigilance (induce higher physical activity and shorter response times; de Wijk and Zijlstra, 2012). Further, olfactory stimuli were often shown to influence mood (Villemure and Bushnell, 2007; de Wijk and Zijlstra, 2012) or stress response (Ludvigson and Rottman, 1989). Environmental odors can also evoke memories (Ehrlichman and Halpern, 1988). Further, olfaction plays an important role in interpersonal communication. Based on body odor, humans can assess some personality traits of other people (Sorokowska et al., 2012), and body odor pleasantness interacts with genetic information significant in mate selection (Milinski et al., 2013). Overall, olfactory processing is significant in many aspects of human life, and it is important to explore individual characteristics affecting olfactory abilities.

From the very beginning of research on the human sense of olfaction, scientists investigated sex differences with this regard. Early studies indicated that females' odor detection, identification and discrimination abilities were better than these of males' (Toulouse and Vaschide, 1899). Studies aimed specifically at testing intersexual differences in olfaction generally obtained results in favor of women (e.g., Koelega and Köster, 1974; Cain, 1982; Doty et al., 1985a). A similar pattern of findings was reported in one of the largest olfactory endeavors conducted so far, the “Smell Survey” involving 1.5 million people whose sense of smell was tested in collaboration with the National Geographic Society (Wysocki and Gilbert, 1989; Corwin et al., 1995). In recent years, however, the question of intersexual differences in odor perception seemed to have been losing scholars' interest and the conviction that female olfaction is better than male became a sort of established knowledge. The view that women's olfactory abilities outperform men's is taken for granted so far that reviews do not focus on existence of such a difference, but rather try to determine its cause (Brand and Millot, 2001; Doty and Cameron, 2009).

However, some studies involving large samples of participants suggested that smell detection ability (Kern et al., 2014) or olfactory identification (Sorokowska et al., 2015b) are similar between the sexes. For example, in a representative sample from Dortmund city (Germany) Vennemann et al. (2008) found more anosmic men than women but they did not find any sex-related differences in olfactory performance among normosmic participants. Additionally, men and women participate in hundreds of studies on olfactory skills which do not address sex differences directly. In the majority of such papers, the authors do not report sex differences, or even, in the vast majority of the articles (see the methods section) they do not present results for male and female participants separately (which may suggest that they do not obtain any significant sex differences). This observation indicates that assumed female superiority in olfactory skills should be treated with caution.

To better understand the potential link between sex and the sense of smell, we should first outline the most important aspects of olfactory performance. In numerous scientific studies, olfactory performance is operationalized as olfactory identification, discrimination, and/or odor detection threshold. They are briefly introduced below.

### Olfactory Identification

Odor identification might be defined as an ability to recognize and name a smell. Odor identification is the most commonly used method of measuring olfactory function in various scientific studies (e.g., Doty et al., 1984a,b; Hummel et al., 1997, 2007). Identification may be assessed in an uncued task, where no retrieval support is provided (free identification) or by cued identification, where a number of alternatives is provided, of which one is the name of the target odor. Performance in odor identification is associated with verbal abilities (Larsson et al., 2004) and cultural context, such that tests need to be specifically adapted for various countries and cultures (e.g., Sorokowska et al., 2014; Oleszkiewicz et al., 2016). Previously published studies present contradictory results about female superiority in this olfactory skill (see e.g., Yang et al., 2010).

### Olfactory Discrimination

Assessment of olfactory discrimination ability is often based on a task where subjects are confronted with a pair or three smells; the participants are to decide whether the two odors are different or which of the three odors is different (Frijters et al., 1980; Potter and Butters, 1980; Hummel et al., 1997, 2007). In the context of the current study it is important that even if odor discrimination seems to be a non-verbal task (as no verbal labels are presented or required), it is to some extent dependent on culture, probably via familiarity effects (Thomas-Danguin et al., 2001; Sorokowska et al., 2014). Also in this test, data are not consistent in terms of potential sex differences (Hummel et al., 2007; Yang et al., 2010).

### Olfactory Threshold

Olfactory threshold can be defined as the lowest concentration at which the presence of an odorant is reliably detected (Hummel et al., 1997, 2007). The term “olfactory detection threshold” refers to the ability to detect odorants; it is often referred to as “overall smell sensitivity.” As compared with higher-order olfactory tasks (e.g., odor identification) measurement of detection thresholds pose few demands on cognitive function (Hedner et al., 2010; Sorokowska et al., 2013). It is believed that measurement of odor thresholds is independent from cultural context and that tests involving this task do not need to be adapted for various countries and cultures (Hoshika et al., 1994; Sorokowska et al., 2013). Also in this test, data about female superiority in olfactory skills are not consistent (Hummel et al., 2007; Yang et al., 2010).

### Sex Differences in Human Olfaction

There are several, non-exclusive elements that could potentially generate sex differences in the presented olfactory abilities. These include, e.g., neuroendocrine, social, and cognitive factors. Below, we briefly introduce and discuss these groups of factors in the context of previous studies on olfactory perception.

First reason of possible female superiority in olfactory perception is associated with neuroendocrine agents, and complex interactions between hormones and olfactory system (Koelega and Köster, 1974; Doty and Cameron, 2009). Although the influence of circulating concurrent levels of gonadal hormones on olfactory function is rather not direct (Doty and Cameron, 2009), numerous studies observed different links between hormones and the sense of smell. (Koelega and Köster, 1974) suggested that sex differences are largest for odors such as androstenone and musks that might be considered biologically meaningful (although the same author presented opposite results in a different study; Koelega, 1994). Additionally, threshold-level sensitivity to certain odors might be associated with menstrual cycle-related fluctuations (e.g., Le Magnen, 1952; Koster and Koelega, 1976; Caruso et al., 2001; Novákov et al., 2014a). Similarly, such threshold-level olfactory sensitivity to specific stimuli (especially socially relevant smells) seems to be increased as a result of female sex hormones, e.g., in late pregnancy (but see: Laska et al., 1996; Ochsenbein-Kölble et al., 2007) or after estrogen injections (Schneider et al., 1958; Good et al., 1976; although these findings were sometimes not replicated). However, sex differences in smell sensitivity are observed also among children (Schriever et al., 2018), which makes the potential conclusions far more complex. In their review on sex differences in olfactory function, Doty and Cameron (2009) suggest that the female superiority observed in olfactory processing might result from an interaction between early endocrine-related influences on regions responsible for smell perception in the human brain and hormonal mechanisms affecting olfactory perception in adult life.

Second, olfactory performance might also depend on olfactory expertise resulting from increased odor awareness. Even in the case of newborns, female babies show more interest in olfactory cues (Schaal et al., 1998). Odor awareness is linked to female-stereotyped activities in childhood and adulthood (Novákov et al., 2014b). Relatedly, performance in memory-related olfactory tasks, like odor identification, can rely on prior exposure to and familiarity with the target odors (Öberg et al., 2002; Cornell Kärnekull et al., 2015). Hence, stimuli used in olfactory identification tests might actually foster womens' performance. Such tests are meant to include only odors of highly familiar items (Hummel et al., 1997, 2007). As women exhibit higher olfactory awareness (Herz and Inzlicht, 2002; Havlicek et al., 2008), they probably pay attention and memorize odors of these familiar items more frequently than men (Smeets et al., 2008). Studies show that indeed, women are more prone to an increase in sensitivity to certain odorants as a result of exposure to these smells (Dalton et al., 2002; Boulkroune et al., 2007). Also, in most countries women still spend more time preparing food than men (GfK, 2014), and many odor identification tasks involve food-related odors.

Third, some olfactory abilities, like e.g., odor identification, are associated with semantic memory and relate to general semantic knowledge, or verbal fluency (Larsson et al., 2000, 2004; Hedner et al., 2010). Perhaps, the sex differences in olfaction (especially olfactory identification) actually stem from lower verbal skills in men, which make it easier for women to find correct verbal labels and answer the questions correctly. Indeed, female olfactory superiority was often observed in tasks involving verbal components (Larsson et al., 2000, 2004; Öberg et al., 2002), and studies suggest that better performance of women in episodic olfactory memory tasks is mediated by their higher proficiency in odor identification (Öberg et al., 2002).

Final group of factors to consider is health-related. First, men are usually more prone to occupational exposure to industrial chemicals and other harmful substances (e.g., cadmium, soot) which are related to olfactory impairment (e.g., Schwartz et al., 1989; Rose et al., 1992; Corwin et al., 1995). However, in this context, the sex difference should be observed mostly for threshold tests, whereas the performance in supra-threshold olfactory tasks, like identification or discrimination, should not be affected to this extent. Further, if olfaction weakens as a result of aging (Kovács, 2004) and males generally age faster than females (Celermajer et al., 1994; Blagosklonny, 2010), olfactory abilities should decrease with age more explicitly in males.

Nevertheless, careful scrutiny of the putative factors shaping sex differences in odor perception indicates several contradictions regarding expected performance of men and women in different types of tests and in different age groups. If some of the arguments presented above are true, then the expected differences should be observable to various extent in identification, discrimination, and threshold tests. The goal of the current metanalysis was to summarize previous results in the extant literature on olfactory performance in relation to sex and to determine whether, and if so in which aspects exactly, female odor abilities are higher than male. To address this question, we performed a meta-analysis on two most commonly used, standardized tests of olfactory function—Sniffin' Sticks Test (SST) (Hummel et al., 1997, 2007) and University of Pennsylvania Smell Identification Test (UPSIT) (Doty et al., 1984a,b).

The Sniffin' Sticks test (SST; Burghardt, Wedel, Germany) is a validated psychophysical tool which allows for complete assessment of the individual's odor perception (Hummel et al., 1997) by pen-like odor dispensers. The test is based on 3 subtests resulting in 4 scores: threshold score (tested by either n-butanol or Phenylethyl alcohol in 16 different concentrations), identification score (16 odorants), discrimination score (16 sets of 3 odorants, out of which two are identical and one is different); a sum of scores of these subtests is a global olfactory score (threshold-discrimination-identification score; TDI). The test has been validated in a number of countries (e.g., Konstantinidis et al., 2008; Tekeli et al., 2015; Oleszkiewicz et al., 2016; Ribeiro et al., 2016) and is widely used in scientific studies.

The University of Pennsylvania Smell Identification Test (UPSIT; Sensonics, Inc., Haddon Heights, NJ) consists of 40 odorants (Doty et al., 1984a,b, 1985b). In order to perform the test, the subject is required to scratch and sniff the odor strip, and choose a correct label from a list of 4 alternatives for each odor. The test was initially standardized in the US population, and adapted versions of UPSIT have been implemented in a number of countries (e.g., Silveira-Moriyama et al., 2010; Yücepur et al., 2012; Fornazieri et al., 2013; Yu and Wu, 2014; Altundag et al., 2015; Li et al., 2015; Jiang and Liang, 2016).

Although numerous tests of olfactory function exist, we focused only on the two mentioned above, as other smell tests are rarely used in more than a few dozen studies. Further, tools other than SST or UPSIT test numerous different aspects of the sense of smell, and in large majority they do not have norms or cultural adaptations. These two factors largely decrease possible chances to compare their results by means of a meta-analysis.

## Materials and Methods

### Search Strategies

We conducted an extensive literature search to identify empirical studies on human olfaction that would employ either UPSIT (Doty et al., 1984a,b) or SST (Hummel et al., 1997, 2007). We searched Google, Google Scholar, Web of Science, Medline, DOAJ, EBSCO, PsycExtra, Academic Search Complete, Health Source: Nursing/Academic Edition, MasterFILE Premier, PsycInfo, PsycArticles, and ERIC databases and used the resources of Elsevier, JSTOR, Science Direct, SAGE Journals, Springer, Taylor & Francis, Wiley, and ProQuest using the following keywords and their combinations: smell^*^, olfact^*^, Sniffin Sticks^*^, SST, UPSIT. We reviewed only articles and research papers written in English. When a full version of an article or statistics about sex differences were not available, we emailed the corresponding authors for provision of the data. The studies found in this phase of the study are presented in Supplementary Materials.

In total, we found 1873 papers, however only 704 with empirical data. 342 studies were further excluded from the current meta-analysis based on different exclusion criteria; the papers could not be included in the meta-analysis: (1) when the sample included exclusively unhealthy people or medical patients (where available, we included the results on healthy controls) (196 studies excluded); (2) when the study was conducted on one sex only (74 studies excluded); (3) when the paper included only people aged below 15 years old (7 studies excluded); (4) when the control sample was subject to placebo manipulation (4 studies excluded); (5) when the original test was modified (46 studies excluded), or (6) when the sample was tested only monorhinally (15 studies excluded). Some excluded studies fulfilled more than one exclusion criterion.

Only 19 articles included necessary data on sex differences. When the paper did not report such statistics, we e-mailed the corresponding author with the request to share the data. In the final analysis we used the data from 82 papers on SST and 24 papers on UPSIT (for the complete list of papers see the Supplementary Materials).

Some papers included in our analyses reported results of several methods of testing (olfactory threshold, discrimination and identification tasks in the SST; Hummel et al., 1997) or reported data of many independent samples. In such cases, the data reported in the study were analyzed separately for each subtest and/or sample. The final samples for olfactory threshold (*SST*) comprised *n* = 8,848 (5,065 women and 3,783 men; 73 independent samples); for discrimination (*SST*): *n* = 8,067 (4,496 women and 3,571 men; 60 independent samples), and for identification (*SST*): *n* = 13,670 (7,501 women and 6,169 men; 77 independent samples). The studies were conducted in at least 24 countries. *For UPSIT*, we retrieved data for a total sample of *n* = 7,154 (3,866 women and 3,288 men; 27 independent samples). The studies were conducted in at least 11 countries.

## Results

As mentioned above, we conducted separate meta-analyses for each aspect of olfaction: threshold, discrimination and identification. All raw data and scripts in Jamovi [https://www.jamovi.org/] used for our analyses can be found under a link https://osf.io/6tfuy/?view_only=9d2ff7b33822417ea82bcc297f8ad13b. Given that olfactory abilities are usually measured using either the SST test (3 subtests) or UPSIT (identification only), we computed four meta-analytical syntheses in total: 3 for each aspect measured by the SST (identification, discrimination, threshold) and 1 for UPSIT (identification). Table 1 presents a summary of effect sizes for sex differences in olfaction across analyzed tests.

**Table 1:** A summary of effect size of sex differences in olfaction across analyzed tests.

**Test**	**Category**	**Effect size for sex differences**				
		**Effect size (Hedges' *g*)**	***SE***	**95% *CI LB***	**95% *CI UB***	***p***
SST	Threshold	0.164	0.033	0.098	0.229	<0.001
	Discrimination	0.109	0.029	0.052	0.165	<0.001
	Identification	0.078	0.033	0.014	0.143	0.017
UPSIT	Identification	0.304	0.046	0.213	0.394	<0.001

*Hedges' gs higher than 0 indicate higher scores of women*.

### SST Threshold Subtest

In the case of odor detection threshold, the meta-analysis of 73 independent samples (total *N* = 8,848) showed a weak effect in favor of women: *g* = 0.164, 95% *CI*: 0.098–0.229, *p* < 0.001. This effect was heterogeneous: *Q* = 110.82, *df* = 72, *p* = 0.002, although the variability was moderate *I*^2^ = 39.38%.

**Figure 1:**
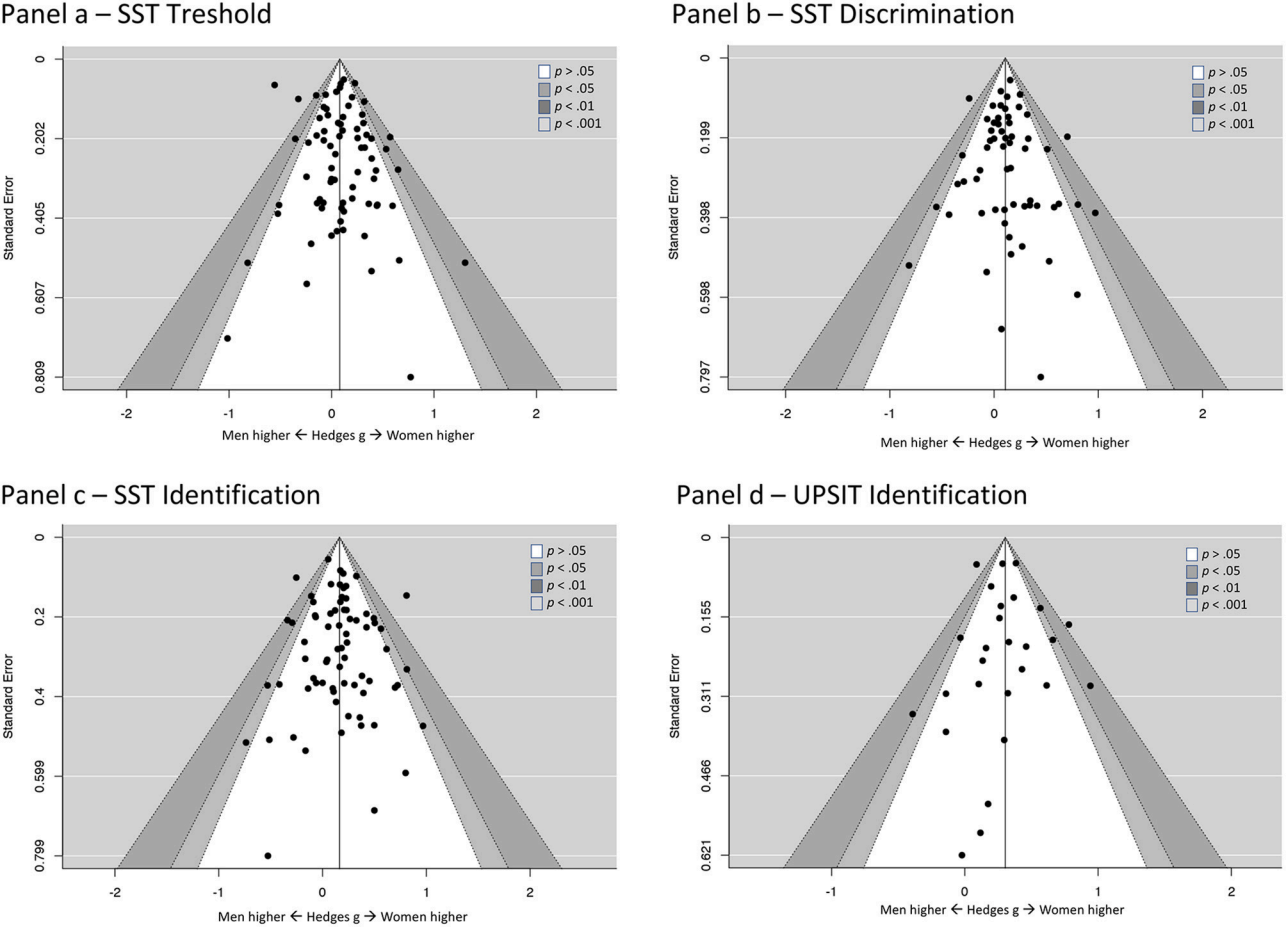
Funnel plots showing lack of publication bias across analyzed tests.

To test whether the obtained effect could be affected by publication bias and selective reporting, we used a funnel plot (Duval and Tweedie, 2000) with two non-parametric techniques to estimate possible bias. Funnel plot (Figure 1, panel c) did not suggest asymmetry (i.e., effects on one side of the funnel did not seem to be regularly suppressed by the effects on the other side). This pattern suggests a lack of publication bias (although such an interpretation is based more on a qualitative judgment, rather than strict statistical rules).

To strengthen the interpretations based on funnel plot, we additionally conducted Egger's regression intercept test (Egger et al., 1997) and Begg and Mazumdar (1994) rank correlation test. Both the regression test (*z* = 0.149, *p* = 0.882) and Begg and Mazumdar (1994) rank correlation test (tau = −0.022, *p* = 0.79), showed no evidence of publication bias.

### SST Discrimination Subtest

The sex differences for SST Discrimination subtest were estimated on 60 independent samples (total *N* = 8,067). The observed sex difference in favor of women was statistically significant (*p* < 0.001), but similar to identification it was very weak according to Cohen's criteria: *g* = 0.109, 95% *CI*: 0.052–0.165. Importantly, this effect was homogeneous across the included studies: *Q* = 71.61, *df* = 59, *p* = 0.126, *I*^2^ = 18.63%.

Inspection of the funnel plot (Figure 1, panel b) did not suggest publication bias. This conclusion was confirmed by non-significant rank correlation test for funnel asymmetry (tau = 0.036, *p* = 0.689) and regression test (*z* = 0.64, *p* = 0.52).

### SST Identification Subtest

There were 77 independent samples (total *N* = 13,670), with SST Identification data available for both men and women. The obtained effect of sex was statistically significant (*p* = 0.017), but very weak: *g* = 0.078, 95% *CI*: 0.014–0.143. In other words, although females did exceed males in terms of identification abilities, the estimated difference was equal to only about 0.08 of standard deviation of identification measures, so should be considered a trivial, even if significant effect. This effect was significantly heterogeneous (*Q* = 199.29, *df* = 76, *p* < 0.001), although the overall level of heterogeneity was moderate (*I*^2^ = 56.76%).

An inspection of the funnel plot (Figure 1, panel a) suggested a lack of publication bias. Similarly, both the regression test (*z* = 1.19, *p* = 0.23) and Begg and Mazumdar (1994) rank correlation test (tau = −0.04, *p* = 0.61), indicated no evidence of publication bias.

### UPSIT

There was a statistically significant, weak-to-medium in size, effect of sex on identification abilities across 27 independent samples that utilized the UPSIT test (total *N* = 7,154). More specifically, females outperformed males of about one-third standard deviation in UPSIT: *g* = 0.304, 95% *CI*: 0.213–0.394, *p* < 0.001. The heterogeneity of reported effects was statistically significant, yet moderate in size: *Q* = 53.995, *df* = 26, *p* = 0.001, *I*^2^ = 53.6%. Also in the case of this test, the funnel plot did not indicate any signs of selective reporting (Figure 1, panel d), similarly as suggested by rank correlation test (tau = −0.18, *p* = 0.20) and regression test for funnel asymmetry (*z* = −0.499, *p* = 0.62).

### Moderator Analysis

The main moderator variable of this meta-analysis was olfactory test: SST vs. UPSIT. As demonstrated in Table 1, 95% confidence intervals around estimated effects did not overlap for identification assessed by means of these two instruments, therefore we concluded that the applied test moderated the obtained effect. Although the effect in both tests was weak, sex differences were more pronounced in UPSIT, than in the SST (*g* = 0.30 for UPSIT vs. *g* = 0.078 for SST identification subtest).

Another tested moderator was average age of samples in included studies. As some studies did not report participants' age, and in some only ranges were given, we dichotomized age variable, so that 0 denoted “younger than 40 years old” and 1 as “40 years old or older” in order to observe whether there are any potential differences between groups containing mostly younger, and mostly older adults. We included this variable into a meta-regression analysis to examine if it differentiated the obtained effect size. Although the age of 40 years is not connected with any particular developmental changes in olfaction (see Sorokowska et al., 2015b), this division allowed us to roughly assess whether age moderated sex differences in olfactory abilities. As illustrated in Table 2, in none of the analyzed cases age moderated the effect size.

**Table 2:** A summary of meta-regression analysis testing moderating effect of participants' age on obtained effect of sex differences in olfaction.

**Test**	**Category**	**k^a^**	**Age**	
			**Estimate (*B*)**	***P***
SST	Discrimination	50	0.02	0.65
	Threshold	70	−0.10	0.17
	Identification	66	−0.10	0.16
UPSIT	Identification	20	0.06	0.60

*Age was introduced as a dichotomized predictor coded 0 = below 40, 1 = 40 or more*.

a *studies which did not provide information about participants' age were excluded from this analysis*.

## Discussion

In the current study, we analyzed extant existing literature to examine whether sex differences in olfaction exist, and more specifically, in which of the following aspects—olfactory identification, olfactory threshold, olfactory discrimination—they may be observed. Furthermore, based on the pattern of results, we aimed to discuss the putative factors shaping sex differences in odor perception. The results of our meta-analysis indicated that women generally outperformed men in olfactory abilities. What is more, they did so in every aspect of olfaction analyzed in the current study. Nevertheless, it needs to be highlighted that although our findings seem to confirm the “common knowledge” on female olfactory superiority, the effect sizes we observed were notably small, especially in comparison with the established intersexual differences in other domains such as risk-taking or attitudes toward sexual intercourses (Byrnes et al., 1999; Petersen and Hyde, 2010). Our data show that sex accounts for 0.15% of variance in the SST identification subtest, 0.30% in SST discrimination subtest, 0.67% in SST threshold subtest and 2.26% in the UPSIT test.

Given the overall pattern of results, different effect sizes observed across SST subtests (which yielded most of the data in the present study) seem particularly interesting in the light of questions on determinants of female olfactory superiority. For example, the effect size for olfactory threshold was over twice as high as the effect size for olfactory identification (SST). Further, the effect size for olfactory discrimination in the SST test was also quite low as compared to the effect for olfactory threshold. This might mean that olfactory threshold tests are the most appropriate to assess sex differences in olfaction. Interestingly, previous studies indicate that both discrimination and identification are sensitive to cognitive factors (Hedner et al., 2010), particularly these associated with semantic memory. At the same time, smell sensitivity as tested by the SST threshold test is believed to be less prone to the influence of verbal components (Hoshika et al., 1994; Sorokowska et al., 2013). Therefore, larger effect size for threshold as compared with identification and discrimination tasks indicates that verbal abilities might have less influence on sex differences in olfactory skills than it was predicted (Lorig, 1999; Larsson et al., 2004; see also: Wallentin, 2009).

Another point worth noting is a considerable difference in effect sizes in olfactory identification between UPSIT and SST. Interestingly, sex differences were more pronounced in UPSIT than in SST identification subtest—the effect size was approximately four times higher in the UPSIT test. It is possible that performance in identification tests is to certain extent determined by the types of odors used in the assessment tools, and these odors might not be gender neutral. Although certain smells are believed to be “typically male,” performance of men in identifying these odors is not better than that of females (Cain, 1982). However, in the context of our findings, this might mean that SST identification subtest contains more odors that are easily identifiable for both sexes, consequently yielding a smaller effect size than UPSIT test in our meta-analysis. One aspect of future research with this regard could be an attempt to create an identification test that would comprise as gender-neutral odors as possible. There are also other potential sources of differences we observed between UPSIT and SST. Performance in identification tests can be very sensitive to even small modifications of the procedure. For example, more contrasted distractors improve the identification test results (Gudziol and Hummel, 2009), higher number of options to choose from decrease the performance (Negoias et al., 2010), and even presenting potential labels before or after smelling an odor significantly influences the identification test score (Sorokowska et al., 2015a). Men were also shown to perform better in olfactory tests when they are provided with help in retrieval of odor names (Cain, 1982). It is therefore possible that, as compared with UPSIT test, alternative response options provided in the SST identification test make execution of this test easier for men. Nevertheless, it needs to be remembered that all these explanations are hypothetical and they need to be explored in further studies. One way to address this possibility would be conducting more meta-analytic studies that would also comprise other, less popular olfactory tests containing different odorants/distractors than Sniffin' Sticks and UPSIT, and assess whether this factor modifies the observed gender differences.

On the grounds that men seem to age faster than women (e.g., Blagosklonny, 2010), which is likely to have effect on their olfactory function as aging is associated with decreased olfactory performance (Sorokowska et al., 2015b), we expected to observe greater sex differences in olfactory abilities in older samples. In our analysis, we compared samples with an average age higher and lower than 40 years in order to observe whether there are any potential differences between groups containing mostly younger, and mostly older adults. Contrary to our assumption, there was no effect of age on sex differences with this regard. One explanation could be the considerable change in female endocrine system caused by menopause, which leads to substantial fall in circulating estrogen, one of hormones associated with increased smell sensitivity in women (Schneider et al., 1958; Good et al., 1976). Hence, although in many terms men experience aging faster, it may be that, due to neuroendocrine factors, olfactory abilities of older women are also vulnerable to an age-related decline.

Following this line of thought, neuroendocrine agents seem to be a plausible factor shaping intersexual differences in olfactory abilities. The most pronounced (although still weak) sex differences in olfactory threshold test results speak for it, given that across studies threshold-level olfactory sensitivity was found to be susceptible to the influence of sex hormones (e.g., Good et al., 1976; Ochsenbein-Kölble et al., 2007). Fluctuations in sex hormones were shown to affect the functioning of other sensory systems too, which supposedly is not coincidental but rather serves certain biological roles (Doty and Cameron, 2009). Among putative biological purposes of sex differences in olfaction are smell-based mate selection, as in self-assessment studies women declare olfaction to be the most important sensory cue in lover choice (Herz and Inzlicht, 2002), and an olfactory aided behavioral immune system that protects pregnant woman and her fetus by distal detection of toxins on the basis of their odor (Doty and Cameron, 2009). As much as our study confirms that women are equipped with a slightly better sense of smell than men, the purpose of intersexual differences with this regard remains, however, speculative. As discussed in the introduction, olfactory performance might also depend on olfactory awareness, which seems to be higher in women even in early childhood. The fact that both neuroendocrine and cognitive factors support better olfactory abilities in women hint the special role the sense of smell plays for them. Future studies should address the question of the biological purpose of olfactory sex differences in more detail, even if the effects we observed in the current meta-analysis were very small.

Possibly, the relatively small sex differences we observed in our research might have anatomical background (Martinez et al., 2017). In this context, differences between men and women are not large; for example, women have smaller nose openings but do not differ from men in intranasal volume (Schriever et al., 2013), and there seems to be no major sex-related difference in olfactory gene receptor expression (Verbeurgt et al., 2014). A few important studies tested sex differences in the olfactory bulb, a part of the brain that influences olfactory function, and that is considered to be the most important relay station in odor processing (Buschhüter et al., 2008). Although microcircuitry (number of cells, number of neurons) is less dense in male olfactory bulbs (Oliveira-Pinto et al., 2014), women tend to have smaller olfactory bulbs (Buschhüter et al., 2008).

In summary, our meta-analysis demonstrated that there exist certain sex differences in olfactory performance. Although significant, the effects were notably small and they translate to very low absolute differences in olfactory test performance. Nevertheless, potential sex differences in olfactory abilities have to be taken into account and controlled for in future studies.

## Author Contributions

PS, AS, TH, and MK conceived and designed the study. MMi, MD, and PS collected data. MK analyzed the data. PS, MKM, AS, MK, TH, MMi, and MD wrote the paper.

### Conflict of Interest Statement

The authors declare that the research was conducted in the absence of any commercial or financial relationships that could be construed as a potential conflict of interest.
