# Supplementary material for: Sex Differences in Human Olfaction: A Meta-Analysis
Source: Front Psychol. 2019 Feb 13;10:242. doi: 10.3389/fpsyg.2019.00242 (PMC6381007; doi:10.3389/fpsyg.2019.00242)
Supplement: Supplementary file 1 [file Table_1.DOCX]

**Supplementary materials**

**Articles included in the metanalyses**

**SST**

Abele, M., Riet, A., Hummel, T., Klockgether, T., & Wüllner, U. (2003). Olfactory dysfunction in cerebellar ataxia and multiple system atrophy. *Journal of neurology*, *250*(12), 1453-1455.

Allen, C., Havlíček, J., & Roberts, S. C. (2015). Effect of fragrance use on discrimination of individual body odor. *Frontiers in Psychology*, *6*, 1115.

Bensafi, M., Frasnelli, J., Reden, J., & Hummel, T. (2007). The neural representation of odor is modulated by the presence of a trigeminal stimulus during odor encoding. *Clinical neurophysiology*, *118*(3), 696-701.

Boesveldt, S., Verbaan, D., Knol, D. L., Visser, M., van Rooden, S. M., van Hilten, J. J., & Berendse, H. W. (2008). A comparative study of odor identification and odor discrimination deficits in Parkinson's disease. *Movement Disorders*, *23*(14), 1984-1990.

Cayonu, M., Salihoglu, M., Altundag, A., Tekeli, H., & Kayabasoglu, G. (2014). Grade 4 tonsillar hypertrophy associated with decreased retronasal olfactory function: a pilot study. *European Archives of Oto-Rhino-Laryngology*, *271*(8), 2311-2316.

Chen, W., Chen, S., Kang, W. Y., Li, B., Xu, Z. M., Xiao, Q., ... & Chen, S. D. (2012). Application of odor identification test in Parkinson's disease in China: a matched case-control study. *Journal of the Neurological Sciences*, *316*(1), 47-50.

Heilmann, S., Strehle, G., Rosenheim, K., Damm, M., & Hummel, T. (2002). Clinical assessment of retronasal olfactory function. *Archives of Otolaryngology–Head & Neck Surgery*, *128*(4), 414-418.

Croy, I., Bojanowski, V., & Hummel, T. (2013). Men without a sense of smell exhibit a strongly reduced number of sexual relationships, women exhibit reduced partnership security–a reanalysis of previously published data. *Biological Psychology*, *92*(2), 292-294.

Croy, I., Landis, B. N., Meusel, T., Seo, H. S., Krone, F., & Hummel, T. (2011). Patient adjustment to reduced olfactory function. *Archives of Otolaryngology–Head & Neck Surgery*, *137*(4), 377-382.

Croy, I., Olgun, S., Mueller, L., Schmidt, A., Muench, M., Hummel, C., ... & Hummel, T. (2015). Peripheral adaptive filtering in human olfaction? Three studies on prevalence and effects of olfactory training in specific anosmia in more than 1600 participants. *Cortex*, *73*, 180-187.

Croy, I., Springborn, M., Lötsch, J., Johnston, A. N., & Hummel, T. (2011). Agreeable smellers and sensitive neurotics–correlations among personality traits and sensory thresholds. *PLoS One*, *6*(4), e18701.

Dudley, L., & Stevenson, R. J. (2016). Interoceptive awareness and its relationship to hippocampal dependent processes. *Brain and Cognition*, *109*, 26-33.

Fasunla, A. J., Douglas, D. D., Adeosun, A. A., Steinbach, S., & Nwaorgu, O. G. B. (2014). Effect of strong fragrance on olfactory detection threshold. *Otolaryngology--Head and Neck Surgery*, *151*(3), 438-442.

Frasnelli, J., Livermore, A., Soiffer, A., & Hummel, T. (2002). Comparison of lateralized and binasal olfactory thresholds. *Rhinology*, *40*(3), 129-134.

Frasnelli, J., Lundström, J. N., Boyle, J. A., Djordjevic, J., Zatorre, R. J., & Jones-Gotman, M. (2010). Neuroanatomical correlates of olfactory performance. *Experimental Brain Research*, *201*(1), 1-11.

Gagnon, L., Vestergaard, M., Madsen, K., Karstensen, H. G., Siebner, H., Tommerup, N., ... & Ptito, M. (2014). Neural correlates of taste perception in congenital olfactory impairment. *Neuropsychologia*, *62*, 297-305.

Guarneros, M., Hudson, R., López-Palacios, M., & Drucker-Colín, R. (2015). Reference values of olfactory function for Mexico City inhabitants. *Archives of medical research*, *46*(1), 84-90.

Guarneros, M., Hummel, T., Martínez-Gómez, M., & Hudson, R. (2009). Mexico City air pollution adversely affects olfactory function and intranasal trigeminal sensitivity. *Chemical senses*, *34*(9), 819-826.

Guarneros, M., Ortiz-Romo, N., Alcaraz-Zubeldia, M., Drucker-Colin, R., & Hudson, R. (2013). Nonoccupational environmental exposure to manganese is linked to deficits in peripheral and central olfactory function. *Chemical Senses*, *38*(9), 783-791.

Gudziol, V., Mackuth, D., Hauswald, B., Knothe, J., Scheuch, K., Zahnert, T., & Hummel, T. (2007). Sense of smell in workers exposed to agricultural odours. *Occupational Medicine*, *57*(2), 149-151.

Huart, C., Legrain, V., Hummel, T., Rombaux, P., & Mouraux, A. (2012). Time-frequency analysis of chemosensory event-related potentials to characterize the cortical representation of odors in humans. *PLoS One*, *7*(3), e33221.

Hummel, T., Delwiche, J. F., Schmidt, C., & Hüttenbrink, K. B. (2003). Effects of the form of glasses on the perception of wine flavors: a study in untrained subjects. *Appetite*, *41*(2), 197-202.

Hummel, T., Haenel, T., & Hull, D. (2002). Assessment of pharyngeal sensitivity to mechanical stimuli using psychophysical and electrophysiological techniques. *Pulmonary pharmacology & therapeutics*, *15*(3), 321-325.

Hummel, T., Kobal, G., Gudziol, H., & Mackay-Sim, A. (2007). Normative data for the “Sniffin’Sticks” including tests of odor identification, odor discrimination, and olfactory thresholds: an upgrade based on a group of more than 3,000 subjects. *European Archives of Oto-Rhino-Laryngology*, *264*(3), 237-243.

Hummel, T., Pfetzing, U., & Lötsch, J. (2010). A short olfactory test based on the identification of three odors. *Journal of Neurology*, *257*(8), 1316-1321.

Iaccarino, L., Shoenfeld, N., Rampudda, M., Zen, M., Gatto, M., Ghirardello, A., ... & Doria, A. (2014). The olfactory function is impaired in patients with idiopathic inflammatory myopathies. *Immunologic Research*, *60*(2-3), 247-252.

Klimek, L., Hummel, T., Moll, B., Kobal, G., & Mann, W. J. (1998). Lateralized and bilateral olfactory function in patients with chronic sinusitis compared with healthy control subjects. *The Laryngoscope*, *108*(1), 111-114.

Knecht, M., Lundström, J. N., Witt, M., Hüttenbrink, K. B., Heilmann, S., & Hummel, T. (2003). Assessment of olfactory function and androstenone odor thresholds in humans with or without functional occlusion of the vomeronasal duct. *Behavioral Neuroscience*, *117*(6), 1135.

Kobal, G., Palisch, K., Wolf, S. R., Meyer, E. D., Hüttenbrink, K. B., Roscher, S., ... & Hummel, T. (2001). A threshold-like measure for the assessment of olfactory sensitivity: the “random” procedure. *European archives of oto-rhino-laryngology*, *258*(4), 168-172.

Kollndorfer, K., Jakab, A., Mueller, C. A., Trattnig, S., & Schöpf, V. (2015). Effects of chronic peripheral olfactory loss on functional brain networks. *Neuroscience*, *310*, 589-599.

Konstantinidis, I., Hummel, T., & Larsson, M. (2006). Identification of unpleasant odors is independent of age. *Archives of Clinical Neuropsychology*, *21*(7), 615-621.

Konstantinidis, I., Printza, A., Genetzaki, S., Mamali, K., Kekes, G., & Constantinidis, J. (2008). Cultural adaptation of an olfactory identification test: the Greek version of Sniffin'Sticks. *Rhinology*, *46*(4), 292.

Landis, B. N., Marangon, N., Saudan, P., Hugentobler, M., Giger, R., Martin, P. Y., & Lacroix, J. S. (2011). Olfactory function improves following hemodialysis. *Kidney international*, *80*(8), 886-893.

Landis, B. N., Scheibe, M., Weber, C., Berger, R., Brämerson, A., Bende, M., ... & Hummel, T. (2010). Chemosensory interaction: acquired olfactory impairment is associated with decreased taste function. *Journal of neurology*, *257*(8), 1303-1308.

Lötsch, J., & Hummel, T. (2006). The clinical significance of electrophysiological measures of olfactory function. *Behavioural brain research*, *170*(1), 78-83.

Lötsch, J., Lange, C., & Hummel, T. (2004). A simple and reliable method for clinical assessment of odor thresholds. *Chemical senses*, *29*(4), 311-317.

Lötsch, J., Ultsch, A., & Hummel, T. (2016). How Many and Which Odor Identification Items Are Needed to Establish Normal Olfactory Function?. *Chemical senses*, *41*(4), 339-344.

Lundström, J. N., & Hummel, T. (2006). Sex-specific hemispheric differences in cortical activation to a bimodal odor. *Behavioural brain research*, *166*(2), 197-203.

Lundström, J. N., Seven, S., Olsson, M. J., Schaal, B., & Hummel, T. (2006). Olfactory event-related potentials reflect individual differences in odor valence perception. *Chemical senses*, *31*(8), 705-711.

Lutterotti, A., Vedovello, M., Reindl, M., Ehling, R., DiPauli, F., Kuenz, B., ... & Berger, T. (2011). Olfactory threshold is impaired in early, active multiple sclerosis. *Multiple Sclerosis Journal*, *17*(8), 964-969.

Mahmut, M. K., & Stevenson, R. J. (2012). Olfactory abilities and psychopathy: higher psychopathy scores are associated with poorer odor discrimination and identification. *Chemosensory Perception*, *5*(3-4), 300-307.

Maione, L., Cantone, E., Nettore, I. C., Cerbone, G., De Brasi, D., Maione, N., ... & Macchia, P. E. (2016). Flavor perception test: evaluation in patients with Kallmann syndrome. *Endocrine*, *52*(2), 236-243.

Miyamoto, T., Miyamoto, M., Iwanami, M., Hirata, K., Kobayashi, M., Nakamura, M., & Inoue, Y. (2010). Olfactory dysfunction in idiopathic REM sleep behavior disorder. *Sleep Medicine*, *11*(5), 458-461.

Mueller, C. A., Grassinger, E., Naka, A., Temmel, A. F., Hummel, T., & Kobal, G. (2006). A self-administered odor identification test procedure using the “sniffin'sticks”. *Chemical senses*, *31*(6), 595-598.

Naka, A., Wolf, A., Renner, B., & Mueller, C. A. (2014). A novel device for the clinical assessment of intranasal trigeminal sensitivity. *Annals of Otology, Rhinology & Laryngology*, *123*(6), 428-433.

Nováková, L., Valentová, J. V., & Havlíček, J. (2013). Olfactory performance is predicted by individual sex-atypicality, but not sexual orientation. *PloS one*, *8*(11), e80234.

Oleszkiewicz, A., Pellegrino, R., Pusch, K., Margot, C., & Hummel, T. (2017). Chemical complexity of odors increases reliability of olfactory threshold testing. *Scientific reports*, *7*, 39977.

Oleszkiewicz, A., Taut, M., Sorokowska, A., Radwan, A., Kamel, R., & Hummel, T. (2016). Development of the Arabic version of the “Sniffin’Sticks” odor identification test. *European Archives of Oto-Rhino-Laryngology*, *273*(5), 1179-1184.

Orhan, K. S., Karabulut, B., Keleş, N., & Değer, K. (2012). Evaluation of factors concerning the olfaction using the Sniffin’Sticks test. *Otolaryngology--Head and Neck Surgery*, *146*(2), 240-246.

Ottaviano, G., Staffieri, A., Stritoni, P., Ermolao, A., Coles, S., Zaccaria, M., & Marioni, G. (2012). Nasal dysfunction induced by chlorinate water in competitive swimmers. *Rhinology*, *50*(3), 294-298.

Pacharra, M., Schäper, M., Kleinbeck, S., Blaszkewicz, M., Wolf, O. T., & van Thriel, C. (2016). Stress lowers the detection threshold for foul-smelling 2-mercaptoethanol. *Stress*, *19*(1), 18-27.

Paschen, L., Schmidt, N., Wolff, S., Cnyrim, C., Eimeren, T. V., Zeuner, K. E., ... & Witt, K. (2015). The olfactory bulb volume in patients with idiopathic Parkinson's disease. *European journal of neurology*, *22*(7), 1068-1073.

Ribeiro, J. C., Simões, J., Silva, F., Silva, E. D., Hummel, C., Hummel, T., & Paiva, A. (2016). Cultural Adaptation of the Portuguese Version of the “Sniffin’Sticks” Smell Test: Reliability, Validity, and Normative Data. *PloS one*, *11*(2), e0148937.

Riva, G., Raimondo, L., Ravera, M., Moretto, F., Boita, M., Potenza, I., ... & Garzaro, M. (2015). Late sensorial alterations in different radiotherapy techniques for nasopharyngeal cancer. *Chemical senses*, *40*(4), 285-292.

Roosenboom, J., Saey, I., Peeters, H., Devriendt, K., Claes, P., & Hens, G. (2015). Facial characteristics and olfactory dysfunction: two endophenotypes related to nonsyndromic cleft lip and/or palate. *BioMed research international*, *2015*.

Rupp, C. I., Kurz, M., Kemmler, G., Mair, D., Hausmann, A., Hinterhuber, H., & Fleischhacker, W. W. (2003). Reduced olfactory sensitivity, discrimination, and identification in patients with alcohol dependence. *Alcoholism: Clinical and Experimental Research*, *27*(3), 432-439.

Schaub, F., & Damm, M. (2012). A time-saving method for recording chemosensory event-related potentials. *European Archives of Oto-Rhino-Laryngology*, *269*(10), 2209-2217.

Schmidt, F., Göktas, Ö., Jarius, S., Wildemann, B., Ruprecht, K., Paul, F., & Harms, L. (2013). Olfactory dysfunction in patients with neuromyelitis optica. *Multiple sclerosis international*, *2013*.

Seubert, J., Freiherr, J., Frasnelli, J., Hummel, T., & Lundström, J. N. (2012). Orbitofrontal cortex and olfactory bulb volume predict distinct aspects of olfactory performance in healthy subjects. *Cerebral cortex*, *23*(10), 2448-2456.

Seubert, J., Ohla, K., Yokomukai, Y., Kellermann, T., & Lundström, J. N. (2015). Superadditive opercular activation to food flavor is mediated by enhanced temporal and limbic coupling. *Human brain mapping*, *36*(5), 1662-1676.

Shu, C. H., Hummel, T., Lee, P. L., Chiu, C. H., Lin, S. H., & Yuan, B. C. (2009). The proportion of self-rated olfactory dysfunction does not change across the life span. *American journal of rhinology & allergy*, *23*(4), 413-416.

Sohrabi, H. R., Bates, K. A., Rodrigues, M., Taddei, K., Laws, S. M., Lautenschlager, N. T., ... & Foster, J. K. (2009). Olfactory dysfunction is associated with subjective memory complaints in community-dwelling elderly individuals. *Journal of Alzheimer's Disease*, *17*(1), 135-142.

Sohrabi, H. R., Bates, K. A., Weinborn, M. G., Johnston, A. N. B., Bahramian, A., Taddei, K., ... & Martins, G. (2012). Olfactory discrimination predicts cognitive decline among community-dwelling older adults. *Translational psychiatry*, *2*(5), e118.

Sorokowska, A., & Hummel, T. (2014). Polish version of the Sniffin'Sticks test-adaptation and normalization. *Otolaryngologia polska= The Polish otolaryngology*, *68*(6), 308-314.

Sorokowska, A., Albrecht, E., & Hummel, T. (2015). Reading first or smelling first? Effects of presentation order on odor identification. *Attention, Perception, & Psychophysics*, *77*(3), 731-736.

Sorokowska, A., Schriever, V. A., Gudziol, V., Hummel, C., Hähner, A., Iannilli, E., ... & Hummel, T. (2015). Changes of olfactory abilities in relation to age: odor identification in more than 1400 people aged 4 to 80 years. *European archives of oto-rhino-laryngology*, *272*(8), 1937-1944.

Sorokowska, A., Sorokowski, P., & Frackowiak, T. (2015). Determinants of human olfactory performance: A cross-cultural study. *Science of the Total Environment*, *506*, 196-200.

Sorokowska, A., Sorokowski, P., & Hummel, T. (2014). Cross-cultural administration of an odor discrimination test. *Chemosensory perception*, *7*(2), 85-90.

Stafford, L. D., & Welbeck, K. (2010). High hunger state increases olfactory sensitivity to neutral but not food odors. *Chemical senses*, *36*(2), 189-198.

Stafford, L. D., Fernandes, M., & Agobiani, E. (2012). Effects of noise and distraction on alcohol perception. *Food Quality and Preference*, *24*(1), 218-224.

Stuck, B. A., Frey, S., Freiburg, C., Hörmann, K., Zahnert, T., & Hummel, T. (2006). Chemosensory event-related potentials in relation to side of stimulation, age, sex, and stimulus concentration. *Clinical Neurophysiology*, *117*(6), 1367-1375.

Tavassoli, T., & Baron-Cohen, S. (2012). Olfactory detection thresholds and adaptation in adults with autism spectrum condition. *Journal of autism and developmental disorders*, *42*(6), 905-909.

Tekeli, H., Senol, M. G., Altundag, A., Yalcınkaya, E., Kendirli, M. T., Yaşar, H., ... & Hummel, T. (2015). Olfactory and gustatory dysfunction in Myasthenia gravis: A study in Turkish patients. *Journal of the neurological sciences*, *356*(1), 188-192.

Tonacci, A., Billeci, L., Tartarisco, G., Mastorci, F., Borghini, A., Mrakic-Sposta, S., ... & Guido, G. (2016). A novel application for cognitive evaluation in mountain ultramarathons: olfactory assessment. *Wilderness & environmental medicine*, *27*(1), 131-135.

Tonacci, A., Borghini, A., Mercuri, A., Pioggia, G., & Andreassi, M. G. (2013). Brain-derived neurotrophic factor (Val66Met) polymorphism and olfactory ability in young adults. *Journal of biomedical science*, *20*(1), 57.

Toussaint, N., de Roon, M., van Campen, J. P., Kremer, S., & Boesveldt, S. (2015). Loss of olfactory function and nutritional status in vital older adults and geriatric patients. *Chemical senses*, *40*(3), 197-203.

Van Thriel, C., Kiesswetter, E., Schäper, M., Juran, S. A., Blaszkewicz, M., & Kleinbeck, S. (2008). Odor annoyance of environmental chemicals: sensory and cognitive influences. *Journal of Toxicology and Environmental Health, Part A*, *71*(11-12), 776-785.

Van Thriel, C., Schäper, M., Kiesswetter, E., Kleinbeck, S., Juran, S., Blaszkewicz, M., ... & Brüning, T. (2006). From chemosensory thresholds to whole body exposures—experimental approaches evaluating chemosensory effects of chemicals. *International archives of occupational and environmental health*, *79*(4), 308-321.

Weierstall, R., & Pause, B. M. (2012). Development of a 15-item odour discrimination test (Düsseldorf Odour Discrimination Test). *Perception*, *41*(2), 193-203.

Welge–Lüssen, A., Wille, C., Renner, B., & Kobal, G. (2003). Test–retest reliability of chemosensory evoked potentials. *Journal of clinical neurophysiology*, *20*(2), 135-142.

Yang, L., Wei, Y., Yu, D., Zhang, J., & Liu, Y. (2010). Olfactory and gustatory function in healthy adult Chinese subjects. *Otolaryngology—Head and Neck Surgery*, *143*(4), 554-560.

Zucco, G. M., Hummel, T., Tomaiuolo, F., & Stevenson, R. J. (2014). The influence of short-term memory on standard discrimination and cued identification olfactory tasks. *Journal of neuroscience methods*, *222*, 138-141.

**UPSIT**

Altundag, A., Tekeli, H., Salihoglu, M., Cayonu, M., Yasar, H., Kendirli, M. T., & Saglam, O. (2015). Cross-culturally modified University of Pennsylvania smell identification test for a Turkish population. *American journal of rhinology & allergy*, *29*(5), e138-e141.

Bergman, J. E., Bocca, G., Hoefsloot, L. H., Meiners, L. C., & van Ravenswaaij-Arts, C. M. (2011). Anosmia predicts hypogonadotropic hypogonadism in CHARGE syndrome. *The Journal of pediatrics*, *158*(3), 474-479.

Bohnen, N. I., Müller, M. L., Kotagal, V., Koeppe, R. A., Kilbourn, M. A., Albin, R. L., & Frey, K. A. (2010). Olfactory dysfunction, central cholinergic integrity and cognitive impairment in Parkinson’s disease. *Brain*, *133*(6), 1747-1754.

Brewer, W. J., Pantelis, C., Anderson, V., Velakoulis, D., Singh, B., Copolov, D. L., & McGorry, P. D. (2001). Stability of olfactory identification deficits in neuroleptic-naive patients with first-episode psychosis. *American Journal of Psychiatry*, *158*(1), 107-115.

Doty, R. L., Applebaum, S., Zusho, H., & Settle, R. G. (1985). Sex differences in odor identification ability: a cross-cultural analysis. *Neuropsychologia*, *23*(5), 667-672.

Economou, A. (2003). Olfactory identification in elderly Greek people in relation to memory and attention measures. *Archives of gerontology and geriatrics*, *37*(2), 119-130.

Fornazieri, M. A., Doty, R. L., Santos, C. A. D., Pinna, F. D. R., Bezerra, T. F. P., & Voegels, R. L. (2013). A new cultural adaptation of the University of Pennsylvania Smell Identification Test. *Clinics*, *68*(1), 65-68.

Fornazieri, M. A., Santos, C. A. D., Bezerra, T. F. P., Pinna, F. D. R., Voegels, R. L., & Doty, R. L. (2014). Development of normative data for the Brazilian adaptation of the University of Pennsylvania Smell Identification Test. *Chemical senses*, *40*(2), 141-149.

Frasnelli, J., Hummel, T., Berg, J., Huang, G., & Doty, R. L. (2011). Intranasal localizability of odorants: influence of stimulus volume. *Chemical senses*, *36*(4), 405-410.

Frasnelli, J., Livermore, A., Soiffer, A., & Hummel, T. (2002). Comparison of lateralized and binasal olfactory thresholds. *Rhinology*, *40*(3), 129-134.

Gill, K. E., Evans, E., Kayser, J., Ben-David, S., Messinger, J., Bruder, G., ... & Corcoran, C. M. (2014). Smell identification in individuals at clinical high risk for schizophrenia. *Psychiatry research*, *220*(1), 201-204.

Ishizuka, K., Tajinda, K., Colantuoni, C., Morita, M., Winicki, J., Le, C., ... & Cascella, N. G. (2010). Negative symptoms of schizophrenia correlate with impairment on the University of Pennsylvania smell identification test. *Neuroscience research*, *66*(1), 106-110.

Jiang, R. S., & Liang, K. L. (2016). Establishment of olfactory diagnosis for the traditional Chinese version of the University of Pennsylvania Smell Identification Test. In *International forum of allergy & rhinology* (Vol. 6, No. 12, pp. 1308-1314).

Jiang, R. S., Kuo, L. T., Wu, S. H., Su, M. C., & Liang, K. L. (2014). Validation of the applicability of the traditional Chinese version of the University of Pennsylvania Smell Identification Test in patients with chronic rhinosinusitis. *Allergy & Rhinology*, *5*(1), e28.

Jiang, R. S., Su, M. C., Liang, K. L., Shiao, J. Y., Wu, S. H., & Hsin, C. H. (2010). A pilot study of a traditional Chinese version of the University of Pennsylvania Smell Identification Test for application in Taiwan. *American journal of rhinology & allergy*, *24*(1), 45-50.

Lafaille-Magnan, M. E., Poirier, J., Etienne, P., Tremblay-Mercier, J., Frenette, J., Rosa-Neto, P., ... & PREVENT-AD Research Group. (2017). Odor identification as a biomarker of preclinical AD in older adults at risk. *Neurology*, *89*(4), 327-335.

Li, K. Y., Fu, H. W., Chen, R. S., Yau, T. Y., & Wu, C. L. (2015). The use of the traditional Chinese version of the University of Pennsylvania Smell Identification Test and the Smell Threshold Test for healthy young and old adults in Taiwan. *Perceptual and motor skills*, *120*(3), 928-943.

Miyamoto, T., Miyamoto, M., Iwanami, M., & Hirata, K. (2010). Olfactory dysfunction in Japanese patients with idiopathic REM sleep behavior disorder: comparison of data using the university of Pennsylvania smell identification test and odor stick identification test for Japanese. *Movement Disorders*, *25*(10), 1524-1526.

Segal, N. L., Topolski, T. D., Wilson, S. M., Brown, K. W., & Araki, L. (1995). Twin analysis of odor identification and perception. *Physiology & behavior*, *57*(3), 605-609.

Shah, M., Muhammed, N., Findley, L. J., & Hawkes, C. H. (2008). Olfactory tests in the diagnosis of essential tremor. *Parkinsonism & related disorders*, *14*(7), 563-568.

Silveira-Moriyama, L., Azevedo, A., Ranvaud, R., Barbosa, E. R., Doty, R. L., & Lees, A. J. (2010). Applying a new version of the Brazilian-Portuguese UPSIT smell test in Brazil. *Arquivos de neuro-psiquiatria*, *68*(5), 700-705.

Weierstall, R., & Pause, B. M. (2012). Development of a 15-item odour discrimination test (Düsseldorf Odour Discrimination Test). *Perception*, *41*(2), 193-203.

Yu, C. Y., & Wu, R. M. (2014). Application of the University of Pennsylvania Smell Identification Test (traditional Chinese version) for detecting olfactory deficits in early Parkinson's disease in a Taiwanese cohort. *Journal of Parkinson's disease*, *4*(2), 175-180.

Yücepur, C., Ozücer, B., Değirmenci, N., Yıldırım, Y., Veyseller, B., & Ozturan, O. (2012). University of Pennsylvania smell identification test: application to Turkish population. *Kulak Burun Bogaz Ihtis Derg*, *22*(2), 77-80.
